# Assessing midwifery services in Iran via the balanced scorecard framework

**DOI:** 10.1093/heapol/czad110

**Published:** 2023-11-10

**Authors:** GholamReza Rezaei, Mohammad SadeghzadehMaharluie, Maedeh Ebrahimi, Marziyeh Ebrahimi

**Affiliations:** Department of Accounting, Faculty of Management and Economics, University of Sistan and Baluchestan, Zahedan, Sistan and Baluchestan Province 98167-45845, Iran; Department of Accounting, Faculty of Economics, Management and Social Sciences, Shiraz University, Shiraz, Fars 71946-84334, Iran; Department of MBA, Apadana Institute of Higher Education, Shiraz, Fars 71946-44635, Iran; Bachelor Student of Midwifery, Islamic Azad University, Arsanjan Branch, Arsanjan, Fars 71946-44635, Iran

**Keywords:** Midwifery, information, management accounting, job satisfaction, balanced scorecard, performance

## Abstract

This study investigates the impact of intra-organizational information, midwife job satisfaction and performance assessment on the quality of midwife services. The questions are empirically tested with survey data obtained from 276 midwives, specialist doctors and nurses, and mothers who recently gave birth in a cross-section of Iranian public healthcare organizations. The results from a structural equation model suggest that an improved performance assessment system leads to higher quality midwife services. In addition, the results indicate that midwife job satisfaction and intra-organizational information increases the quality of midwife services, both directly and indirectly, through the mediating effect of a performance assessment system. Our study contributes to the growing research exploring the interface between accounting and health issues by recognizing the importance of a performance assessment system of midwifery services via the balanced scorecard framework for understanding the quality of midwife services.

Key messages

- The results suggest that an improved performance assessment system leads to higher quality midwife services.
- The results indicate that midwife job satisfaction and intra-organizational information increase the quality of midwife services.
- Our study contributes to the growing research exploring the interface between accounting and health issues.

## Introduction

One of the most important healthcare services in any country is midwifery, which plays an important role in the birth of babies and their health. Indeed, pregnancy and childbirth are important events in the lives of families (Haghdoost *et al.*, 2021). In fact, it is essential to improve the quality of care delivered and provide an appropriate work environment in healthcare organizations. Therefore, paying attention to the quality of services in the midwifery sector is critically important, and studying this is the major purpose of our paper. We believe that first, there must be a performance evaluation system to improve quality, second, information is needed to evaluate performance and service quality, and third, the connection between performance assessment systems and worker satisfaction is crucial and midwives are no exception in this regard. Thus, the major purposes of this study are to investigate: (1) the status of intra-organizational information on midwifery services in Iran; (2) the status of midwife job satisfaction in Iran; (3) the role of management accounting systems in the performance assessment of midwifery services in Iran based on the balanced scorecard framework; (4) the quality of the services in the midwifery sector from the point of view of expectant mothers; (5) the relationship between the status of intra-organizational information of midwifery services, midwife job satisfaction, the role of management accounting systems in the performance assessment of midwifery services based on the balanced scorecard framework and the quality of the services in the midwifery sector from the point of view of expectant mothers in a comprehensive model. As a result, the purpose of our paper is to provide evidence in support of using health systems performance assessment tools to strengthen health systems and create enabling environments for higher quality midwifery care (through improved job satisfaction and intra-organizational information).

Today, favourable performance is crucial for healthcare services. Performance evaluation can be used as a basis for assigning medical staff to a specific position, preparing a reasonable and transparent reward system, establishing fair career development, and evaluating the application of an organization’s resources (Akbar and Sujanto, 2021). Healthcare organizations operate in a complex, competitive and dynamic environment and are incentivized to provide greater flexibility and higher quality of service (Lin *et al.*, 2013). In fact, performance appraisal systems play an important role in healthcare organizations to provide information about the success of the organization as well as its competitive strategies, and ultimately link these strategies to the organization’s activities. Performance measurement systems serve as strategic frameworks for healthcare providers to track progress and evaluate best practices, linking health outcomes with health system strategies and functions. An effective performance evaluation system is essential to control, monitor and improve service quality at medical facilities (Lin *et al.*, 2013). Although the healthcare industry has embraced performance assessment systems in carrying out its duties to provide high-quality healthcare services (Owusu Kwateng *et al.*, 2021), there is little systematic evidence of their use and benefits to midwifery services. Hewitt *et al.* (2021) stated that there is little known about what constitutes the optimal leadership and management of midwifery. In Iran, governmental and private hospitals in cooperation with the Ministry of Health and Medical Education (MOHME) and medical universities are responsible for providing midwifery services. The burning questions that arise here are as follows. What is the status of intra-organizational information in midwifery services in Iran? What is the role of management accounting systems in the performance assessment of midwifery services in Iran based on the balanced scorecard framework? What is the status of midwife job satisfaction in Iran? How good is the quality of the services in the midwifery sector from the point of view of expectant mothers? In addition, what is the relationship between the status of intra-organizational information of midwifery services, midwife job satisfaction, the role of management accounting systems in the performance assessment of midwifery services based on the balanced scorecard framework, and the quality of the services in the midwifery sector from the point of view of expectant mothers?

We believe that it is very important to focus on the performance of hospitals in the area of midwifery services. Global evidence has highlighted the challenges of disrespect and abuse of pregnant women accessing health services (Bohren *et al.*, 2015; Haghdoost *et al.*, 2021). These instances of abuse reduce families’ trust in the healthcare system and in providers such as midwives. It is important to consider the factors that contribute to disrespect and abuse, such as health system constraints, burnout and poor job satisfaction. Job satisfaction has been defined as a pleasurable or positive emotional state resulting from the appraisal of one’s job or job experiences and is described as a comprehensive concept involving the following aspects: job scope including demands and tasks, social relevance and prospects for career growth, workplace relationships, teamwork, support from peers and supervisors, work organization, working hours, working environment and job security (Badr *et al.*, 2013; Hotchkiss *et al.*, 2015; Weisman and Nathanson, 1985).

Low job satisfaction is associated with higher levels of employee dissent (Staw, 1984). Since the use of any system (e.g. the performance evaluation system) requires the support of the users of that system (Burney and Swanson, 2010; Alipour *et al.*, 2023; Dorta-Afonso *et al.*, 2023), and this support requires the satisfaction of the employees with their jobs (Burney and Swanson, 2010), we believe the job satisfaction of midwives can have an affect on the effectiveness of the hospitals’ performance evaluation system. The evidence of Burney and Swanson (2010) shows that there is a positive relationship between job satisfaction and the performance assessment system based on the balanced scorecard. Also, the absence of job satisfaction among healthcare’s labour can affect their practice, which in turn can affect patients’ satisfaction directly or indirectly (Zangaro and Soeken, 2007). This observation has made job satisfaction a global concern (Salahat and Al-Hamdan, 2022).Unlike in other countries (Watson *et al.*, 1999; Skinner *et al.*, 2012; Warmelink *et al.*, 2015), the job satisfaction of midwives in Iran is not at a suitable level (Hadizadeh Talasaz *et al.*, 2014; Khavayet *et al.*, 2018), and there are concerns globally that the number of midwives will not be adequate for future staffing level requirements (Sheehy *et al.*, 2021). In addition, de Jonge *et al.* (2015) showed that to achieve primary care for all childbearing women and infants, hospitals need tools to monitor the performance of primary midwifery care. For this, the hospitals need information, which requires a good information system. In other words, the requirement to evaluate the performance of each department demands the existence of information and an efficient information system.

Intra-organizational information is information that is prepared and reported by the management accounting unit for internal users (Namazi and Rezaei, 2023). The management accounting unit in the treatment system of each country is responsible for preparing and providing information to control and manage diseases and treatment (as part of intra-organizational information) (Rezaei *et al.*, 2022). Intra-organizational information can improve the quality of patient care by increasing the timely access to and accuracy of clinical and administrative information related to the patient and its exchange between employees and departments within the hospital (Alipour *et al.*, 2023). Intra-organizational information is related to performance assessment. Tahar and Ménacère (2013) found that part of the intra-organizational information in the governmental sector (e.g. healthcare organizations) revolves around organizational performance evaluation and its improvement. In MacAulay *et al.*’s (2019) opinion, assessment of the outcomes of any programme is essential because it demonstrates the success or failure of the programme. However, traditional and one-dimensional performance assessment systems are not very effective, especially for healthcare services. The evidence from previous research also supports this issue (Baker and Pink, 1995; Kaplan *et al.*, 2001; Kaplan and Norton, 2006; Qingwei, 2012; Zhijun *et al.*, 2014; Alharbi *et al.*, 2016). Considering the disadvantages of one-dimensional performance assessment systems, Kaplan and Norton (1992) presented a system called the ‘balanced scorecard’ for performance assessment. Using this system in hospitals and healthcare organizations can be very beneficial for managers. Therefore, in this article, we intend to investigate the performance assessment of one of the most important services of hospitals using the comprehensive framework of the balanced scorecard.

The balanced scorecard system was first introduced in 1992 by Kaplan and Norton, and since then it has been widely used in various industries and sectors (e.g. Elbanna *et al*. 2022). The field of healthcare organizations has been not exempt from this issue (e.g. Lin *et al.* 2013; Zhijun *et al.* 2014; Alharbi *et al.* 2016). This system is a tool to improve managerial insight into organizational performance (Tawse and Tabesh, 2023) and evaluates performance based on four perspectives: financial, patients and society, learning and development, and intra-organizational processes (Kaplan and Norton, 1992; 2006; Kaplan *et al.*, 2001; Rezaei *et al.*, 2022). Based on Kaplan and Norton (1992), the financial performance perspective indicates whether the organization’s strategy, implementation and execution are contributing to bottom-line improvement. The patients and society perspective demands that managers translate their general mission statement on patient service into specific measures that reflect the factors that really matter to patients. The intra-organizational processes perspective also stems from the organizational processes that have the greatest impact on patient satisfaction. The learning and development perspective describes how the people, technology and organizational climate combine to support strategy. Measures in this perspective are lead indicators for improvements in the intra-organizational processes, patients and society, and financial perspectives. From the viewpoint of De Geuser *et al.* (2009), the value of the balanced scorecard system was found to derive from three sources: (1) it helped translate strategic goals into action; (2) it positively influenced managerial practices on a continuous basis; and (3) it helped to align resources and strategic objectives. Therefore, it can be expected that the existence of a suitable performance assessment system (such as the balanced scorecard system) will help to improve the quality of services.

Based on the general connotations of job satisfaction theories and the strategic management accounting literature (Fuertes *et al.*, 2020; Dorta-Afonso *et al.*, 2023), we posit that the effect of midwife job satisfaction and intra-organizational information on the quality of the services in the midwifery sector is direct; and that a part of the effect is indirect and mediated by the existence of a suitable performance assessment system. The mediation relations are asserted according to the mediating literature (Baron and Kenny, 1986) to explicate how the relationship between midwife job satisfaction and intra-organizational information and quality of the services is formed.

Our paper makes a three-way contribution to the field. Initially, we document novel evidence for the quality of the services in the midwifery sector from the point of view of expectant mothers in Iran as a developing country. Every day, ∼810 women die from pregnancy-related causes, most of which are preventable and happen in low- and middle-income countries. Therefore, based on the fact that midwives play a significantly critical role in providing primary care for saving the lives of mothers and babies (Khosravi *et al.*, 2022), it is very important to investigate the quality of the services in the midwifery sector.

Second, we contribute to the literature by identifying the mechanisms by which management accounting systems, through performance assessment of midwifery services and intra-organizational information, effect the quality of the services in the midwifery sector. This issue is very important as an interdisciplinary discussion in midwifery. Ultimately, our paper enriches the literature on the quality of the services in the midwifery sector with regard to psychological issues. Overall, the evidence of our paper may be valuable for producing decisions about the use of performance assessment systems to improve the quality of midwifery services in other countries.

## Materials and methods

### Design, population and sample

**Figure 1.**
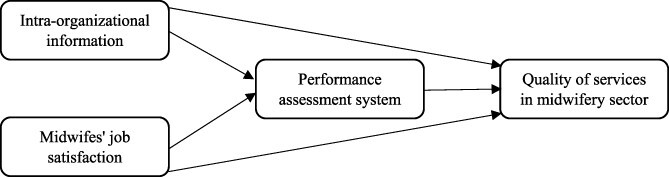
Theoretical model

The purpose of our research is to investigate the quality of services in the midwifery sector from the point of view of expectant mothers, midwife job satisfaction, the status of intra-organizational information of the midwifery sector, and the role of management accounting systems in the performance assessment of midwifery services in Iran’s healthcare centres from the perspective of people working in this sector. In this regard, the research method is exploratory, survey type and a cross-sectional study. Based on the literature presented, we propose that the existence of a good performance evaluation system for the midwifery department and improved job satisfaction for midwives can lead to better quality midwifery services. Figure 1 shows the theoretical model of the study, selected variables and their causal relationships; it provides a basis to investigate how the quality of midwifery services is affected by the designated variables.

In our model, based on the mediating literature framework of Baron and Kenny (1986), the variable of performance assessment of midwifery services in Iran based on the balanced scorecard framework was selected as a mediating variable, which can effect the quality of midwifery services (Lin *et al.*, 2013) and is affected by midwife job satisfaction and intra-organizational information (Zangaro and Soeken, 2007; Burney and Swanson, 2010). In addition, based on Fig. 1, midwife job satisfaction and intra-organizational information are independent variables that directly effect the quality of services in the midwifery sector. These predictions, are based on the general connotations of job satisfaction theories and the strategic management accounting literature (Zangaro and Soeken, 2007; Fuertes *et al.*, 2020; Alipour *et al.*, 2023; Dorta-Afonso *et al.*, 2023).

Due to the significance and extensiveness of health and medical services of governmental hospitals in Iran for midwifery services, and the familiarity of the researchers with the activities of these organizations, governmental hospitals in Iran were selected as the research domain. According to the purpose of our research, the statistical population consists of two parts. Part one consists of all midwives, specialist doctors, nurses and managers who play a role in the birth process of babies (consultation stages during pregnancy, childbirth and postnatal counselling measures) in any manner in the governmental hospitals in Iran. The second part includes mothers who recently gave birth in Iran’s public hospitals. Therefore, data is gathered from Iran’s governmental hospitals. Because of its particular circumstances, Iran is one of the attractive domains for research in the field of analysing the performance of midwifery services. This country has been subject to economic sanctions for many years, which has led to many changes in the method of managers’ decisions in various fields (e.g. how to provide healthcare services). In recent years, also, in Iran most pregnant women tend to receive caesarean section services instead of vaginal delivery for the birth of babies (Azami-Aghdash *et al.*, 2014); this issue indicates that the quality of midwifery services in Iran needs to be investigated. The study employed simple random sampling. In spite of the effort of the researchers via the human resources department of the MOHME, the exact number of the midwives, specialist doctors, nurses and managers who may play a role in the birth process of babies could not be determined. However, the approximate number of midwives and managers who may play a role in the birth process of babies at various levels in the governmental hospitals was considered a large statistical population^1^. Using Morgan’s table for large populations, the sample size consisted of 384 people. However, to obtain superior reliability for the study, and based on the low rate of questionnaire returns, as reported from the preliminary studies and previous research, as well as the lack of accurate statistics in this regard, in 2022 a total of 400 questionnaires were distributed among all members of the first part of the statistical sample (via in-person delivery, email and post). A recall to the respondents who had not answered within 4 weeks was then conducted either in-person, by mail or email, as described by Nguyen *et al.* (2017). Eventually, after removing unsuitable questionnaires, in all 276 (69%) questionnaires were completed and utilized in this study. This response rate is similar to and even better than those reported in other management accounting and performance assessment surveys [e.g. Rezaei *et al.* (2022)]. In addition, it complies with Baruch’s (1999) recommendations in that he reported an average (standard deviation) response rate for surveys of 35.5% (13.3%). For comparison and analysis purposes, the required number of samples of questionnaires on the quality of services in the midwifery sector from the point of view of expectant mothers was considered to be 276. For this purpose, in 2022, the distribution of questionnaires continued hospital-to-hospital in Iran until 276 completed questionnaires were received^2^. Therefore, a total of 552 questionnaires was received from two parts of the statistical population; demographic features related to these two parts of the statistical population are given in panels A and B of Table 1.

**Table 1.** Demographic features of respondents

Panel A The first part of the statistical population: midwives, specialist doctors, nurses and managers who may play a role in the birth process of babies				
Gender	Male		Female	
	16 (5.8%)		260 (94.2%)	
Educational degree	Associate degree	Bachelor’s	Master’s	PhD
	13 (4.7%)	138 (50%)	81 (29.3%)	44 (15.9%)
Age, years	<30	30–40	41–50	>50
	34 (12.3%)	81 (29.3%)	114 (41.3%)	47 (17.1%)
Work experience, years	<5	5–10	11–20	>20
	43 (15.6%)	120 (43.5%)	101 (36.6%)	12 (4.3%)
**Panel B** **The second part of the statistical population: mothers who recently gave birth in Iran’s public hospitals**				
Age, years	<25	25–35	36–45	>45
	33 (12%)	107 (38.8%)	126 (45.6%)	10 (3.6%)
Educational degree	Associate degree and others	Bachelor’s	Master’s	PhD
	34 (12.3%)	90 (32.6%)	122 (44.2%)	30 (10.9%)

### Questionnaire and measures

Data was collected using a questionnaire consisting of 62 questions in four sections. Section one tends to seek information regarding the qualitative features of the management accounting system and the status of intra-organizational information on midwifery services in Iran (8 questions). Section two deals with the role of management accounting systems in the performance assessment of midwifery services in Iran based on the balanced scorecard framework (16 questions). The questions of section two were designed into four perspectives according to the balanced scoreboard system: financial (4 questions), patients and society (4 questions), learning and development (4 questions) and intra-organizational processes (4 questions). These perspectives are important to hospitals because they help hospitals map their projects and initiatives to the different strategic objectives, which in turn ensures that the projects and initiatives are tightly focused on delivering the most strategic objectives. In principle, the questions of the second section determine to what extent the various dimensions of performance are evaluated in Iran’s hospitals for midwifery services; to be more precise, the existence of the performance assessment system and its scope are measured. The third section is related to midwife job satisfaction and consists of 30 questions in 5 parts (including nature of work, salary, supervisor, promotions and colleagues). The first part of the statistical population answers the questions related to these three sections. Section four of our questionnaire is related to quality of services from the point of view of expectant mothers (8 questions) and is answered by the second part of the statistical population.

The questionnaire also included a cover letter explaining the purpose of the study and a preliminary section intended to collect data on the respondents and their organizations. In addition, the survey items scored on a seven-point Likert-type scale ranging from one (strongly disagree) to seven (strongly agree). Appendix 1 illustrates a sample of the questionnaire.

### Data analysis

The present study utilized Cronbach’s alpha and SPSS-22 software to verify the reliability of the questionnaire. The results of testing the questionnaire indicated adequate questionnaire reliability (the status of intra-organizational information of midwifery services, 0.837; midwife job satisfaction, 0.851; the role of management accounting systems in the performance assessment of midwifery services, 0.884, and quality of services, 0.862). It is worth mentioning that the role of a management accounting system in the performance assessment of midwifery services consisted of performance assessment of the financial aspect, the customer (patient) aspect, learning and growth (staff) aspect, and intra-organizational processes aspect with 0.829, 0.905, 0.896 and 0.857 respectively. To analyse the data from the questionnaire, the univariate test of differences in means (one-sample t-test) and structural equation modelling (SEM) using SPSS-22 and Smartpls-2 was employed. An advantage of SEM is that it can simultaneously evaluate the proposed hypotheses and test the measurement reliability (Smith and Langfield-Smith, 2004).

## Results

Analyses began with the demographic features of respondents. The demographic features of respondents are described in Table 1. In our survey, respondents have different ages, work experiences and educational degrees between males and females, and the statistics for each group can be seen in Table 1. Panel A of Table 1 provides the demographic features of respondents of the first part of the statistical population. This indicated that ∼5.8% of the respondents were male and ∼94.2% were female, with a frequency of 16 and 260, respectively. The respondents age shows that 12.3% of them are <30 years old, 29.3% are 30–40, 41.3% are 41–50 and 17.1% are >50 years old. Therefore, the age group between 41–50 has the most respondents. With regard to education, the most common degree among the respondents was a Bachelor’s degree with a frequency of 50%, followed by Master’s and PhDs with 29.3 and 15.9%, respectively. Most respondents, 43.5%, have 5–10 years’ work experience, followed by 36.6% having 11–20 years’ work experience.

Panel B of Table 1 provides the demographic features of respondents of the second part of the statistical population (mothers who recently gave birth in Iran’s public hospitals). The table shows that 12% of mothers are <25 years old, 38.8% are between 25–35, 45.6% are between 36–45 and 3.6% are >45 years old. Therefore, the age group between 36–45 years has the most respondents, indicating that the age of those giving birth in Iran is relatively high. With regard to education for mothers who recently gave birth in Iran’s public hospitals, the most common degree among the respondents was a Master’s degree with a frequency of 44.2% which is followed by Bachelor’s, associate and others, and PhD degrees with 32.6, 12.3, and 10.9%, respectively.

Table 2 reveals the descriptive statistics of the research variables and univariate test of differences in means (one-sample t-test). The significance level of the t-statistic indicates a significant difference between the response of the participants and the tested value (number four; mid-point of a 7-point response) for most variables. For instance, the intra-organizational information of midwifery services is higher than the tested value (number four) and significant. Likewise, the same is true for the quality of services for midwifery services. In addition, the performance assessment of midwifery services in Iran based on the balanced scorecard framework is at an appropriate level. The details of the evaluation performance via balanced scorecard also indicate that the financial, customer (patients), employee, and organizational processes dimensions are above the tested values and are significant. However, Table 2 shows that the job satisfaction of midwives in Iran is not at a suitable level and is the same as the tested value.

**Table 2.** Descriptive statistics of research variables

Variable		Minimum	Maximum	Mean	SD	t-statistic	Significance
Intra-organizational information		2.75	6.63	5.20	0.87	22.74	0.000^a^
Midwife job satisfaction	Nature of work	1.83	6.50	4.22	1.00	3.58	0.000^a^
	Salary	1.33	6.67	4.11	1.04	1.82	0.069
	Supervisor	1.33	6.83	4.31	1.05	4.93	0.000^a^
	Promotions	1.50	6.50	4.09	1.02	1.47	0.142
	Colleagues	1.83	6.50	4.01	1.02	0.31	0.755
	Total score	1.67	5.63	4.15	0.84	2.95	0.003^a^
Performance assessment of midwifery services	Performance assessment of the financial aspect	2.25	6.75	5.21	0.93	21.70	0.000^a^
	Performance assessment of the customer (patient) aspect	1.75	7.00	5.03	1.21	14.27	0.000^a^
	Performance assessment of learning and growth (staff) aspect	2.75	7.00	5.20	0.85	23.24	0.000^a^
	Performance assessment of intra-organizational processes aspect	2.25	7.00	4.94	1.15	13.65	0.000^a^
	Total score	3.00	6.50	5.10	0.69	26.38	0.000^a^
Quality of services		2.00	6.88	4.97	1.00	16.08	0.000^a^

a Statistical significance at the 0.01 level.

The first step in the analysis is to develop a measurement model. This model aims to specify the relationships between observed and latent variables (Black *et al.*, 2010). Our measurement model consists of four theoretical latent factors (intra-organizational information, midwife job satisfaction, performance assessment system and quality of services). The standardized factor loadings and statistics from the measurement analysis are suitable. Model fit is determined by the degree of correspondence between the observed and the estimated covariance matrix. Our measurement model fits well according to goodness-of-fit (Normed Fit Index = 0.94 > 0.90) and badness-of-fit (Standardized Root Mean Square Residual = 0.06 < 0.09) indices [e.g. Arbuckle and Wothke (1999), Bentler and Bonett (1980); Black *et al.* (2010)].

Regarding item reliability, as mentioned by Black *et al.* (2010), significant item loadings >0.50 are sufficient to establish reliability. All items suggest good reliability with all loadings being significant (*P* < 0.05) and >0.50. Next, convergent validity was assessed by examining the composite reliability (CR) and average variance extracted (AVE). As Table 3 confirms, the CR index (rho_a and rho_c) of all constructs is good as all scores equal or exceed the threshold value of 0.70 (Black *et al.*, 2010). Moreover, most AVE scores are close to, or higher than, the threshold value of 0.50, which indicates that at least 50% of the indicator variance is accounted for by construct variance rather than noise (Fornell and Larcker, 1981). In addition, discriminant validity of the constructs was assessed by the Heterotrait-Monotrait ratio of correlation (HTMT) index. As observed from Table 3, the HTMT index scores are <0.9, which demonstrates strong evidence of discriminant validity (Henseler *et al.*, 2015). Overall, the results from the measurement model indicate adequate construct validity for all constructs in the model.

**Table 3.** Construct reliability and validity

					HTMT index		
	Cronbach’s alpha	Composite reliability (rho_a)	Composite reliability (rho_c)	AVE	1	2	3
Intra-organizational information (1)	0.764	0.780	0.829	0.683	–	–	–
Midwife job satisfaction (2)	0.943	0.946	0.948	0.681	0.640	–	–
Performance assessment system (3)	0.826	0.838	0.856	0.580	0.706	0.750	–
Quality of services	0.814	0.850	0.863	0.655	0.623	0.781	0.694

In addition, the correlation analysis in Table 4 indicates a strong relationship between quality of services and intra-organizational information (*r* = 0.719), performance assessment (*r* = 0.778) and midwife job satisfaction (*r* = 0.699). Moreover, we find a significant positive correlation between performance assessment of midwifery services and intra-organizational information (*r* = 0.749) and midwife job satisfaction (*r* = 0.785).

**Table 4.** Correlations matrix

	Intra-organizational information	Midwife job satisfaction	Performance assessment system	Quality of services
Intra-organizational information	1			
Midwife job satisfaction	0.565^a^	1		
Performance assessment system	0.749^a^	0.785^a^	1	
Quality of services	0.719^a^	0.699^a^	0.778^a^	1

a Statistical significance at the 0.01 level.

**Figure 2.**
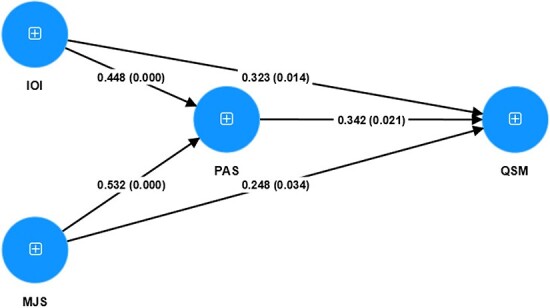
Structural model with path coefficients and significance

Note: IO, intra-organizational information; MJS,midwife job satisfaction; PAS, performance assessment system; QSM, midwifery services quality

To examine our theoretical model, the results presented in Table 5 and Fig. 2 illustrate the significant associations in our path model. Consistent with our theoretical expectations, the path estimate between intra-organizational information of midwifery services and the performance assessment of midwifery services based on the balanced scorecard framework is positive and significant (β = 0.448, *P* < 0.01). Also, linking higher levels of midwife job satisfaction to higher levels of performance assessment of midwifery services based on the balanced scorecard framework is thus theoretically supported. As predicted earlier, higher levels of intra-organizational information are positively associated with the quality of services (β = 0.323, *P* < 0.05). Furthermore, in support of the theoretical prediction, the results indicate that higher levels of midwife job satisfaction are associated with higher levels of quality of services (β = 0.248, *P* < 0.05). In support of Fig. 1, we found a statistically significant positive association between the performance assessment system and the quality of services (β = 0.342, *P* < 0.05).

**Table 5.** Path coefficient

	Path	Coefficient	t-statistic (*P*-value)	Theoretically supported
Direct effects	Intra-organizational information—performance assessment system	0.448	5.146 (0.000*)	Yes
	Midwife job satisfaction—performance assessment system	0.532	8.022 (0.000*)	Yes
	Intra-organizational information—quality of services	0.323	2.466 (0.014^**^)	Yes
	Midwife job satisfaction—quality of services	0.248	2.126 (0.034^**^)	Yes
	Performance assessment system—quality of services	0.342	2.302 (0.021^**^)	Yes
Indirect effects	Intra-organizational information—performance assessment system—quality of services	0.153	2.235 (0.025^**^)	Yes
	Midwife job satisfaction—performance assessment system—quality of services	0.182	2.049 (0.040^**^)	Yes

Note: * and ** indicate statistical significance at the 0.01 and 0.05 level, respectively.

In addition to the direct paths coefficient, in Table 5 and Fig. 2, the coefficients of indirect paths are also shown. To be precise, the performance assessment of midwifery services based on the balanced scorecard framework has a mediating role in the relationship between midwife job satisfaction and quality of services (β = 0.182, *P* < 0.05) and intra-organizational information and quality of services (β = 0.153, *P* < 0.05).

## Discussion

This study was aimed at providing conceptual and empirical evidence on the status of intra-organizational information, a performance assessment system based on the balanced scorecard framework, midwifejob satisfaction and the quality of services in the midwifery sector in Iran, and how performance assessment systems affect the quality of midwifeservices in the public health sectors. By presenting an epistemic conceptual model between the explanatory construct of midwife job satisfaction and intra-organizational information, the explained construct of the quality of midwife services, designating the performance assessment system as the mediating variable, enhanced the theoretical basis of the literature; and then by utilizing SEM, it empirically tested the designated model in the public health ministry of Iran.

The study provided empirical evidence which supports the theoretical basis. The results of the testing show that intra-organizational information and midwife job satisfaction positively affect the quality of midwife services, directly. However, they can also influence the quality of midwife services through the mediation of the performance assessment system based on the balanced scorecard framework. The existence of intra-organizational information increases awareness of managers and, this, in turn, decreases information asymmetry between them (Namazi and Rezaei, 2023). Therefore, intra-organizational information increases and improves based on the principle of fineness (Namazi, 1985). Such improvement would influence users’ behaviour in improved information decision-making and the effect of information on users’ behaviour (Magee, 1986; Church *et al.*, 2019). In effect, the intra-organizational information provides both decision facilitating and decision influencing roles in the performance assessment system and quality of the services in the midwifery sector. In addition, for a system (e.g. performance assessment system) to be effective requires that users support that system, and such support depends on the users satisfaction with their work and the organization (Burney and Swanson, 2010; Alipour *et al.*, 2023; Dorta-Afonso *et al.*, 2023). Therefore, according to the expectation, the job satisfaction of midwives is associated with the proper functioning of the performance assessment system. Also, midwives who are satisfied with their work will try to perform their duties in a better way and therefore the quality of their services will improve. This finding about job satisfaction is consistent with Burney and Swanson (2010). On the other hand, when midwives realize that their performance is evaluated by a system from different perspectives, they will try to perform their duties more efficiently. As a result, the existence of the performance assessment system has improved the quality of midwifery services. In such a situation, according to mediation literature (Baron and Kenny, 1986), the existence of the performance assessment system plays a mediating role in the relationship between intra-organizational information and midwife job satisfaction with regard to the quality of midwifery services in Iran.

## Conclusion

This study has significant implications for healthcare organizations in the public sectors. In general, it provided useful evidence regarding the factors affecting the quality of midwife services in Iran. This research showed that it is possible to improve the status of intra-organizational information, midwife job satisfaction and the performance evaluation system and thus improve the quality of midwife services. In Iran, the MOHME has contributed significantly to the status of information and performance assessment systems by setting up a management accounting unit. This, in turn, has led to an improvement in midwifery services in Iran. In fact, not only doctors and midwives but also accountants (especially management accountants) can play an important role in the quality of midwifery services. They can help hospital managers, doctors and midwives by providing information and guidance and assisting them in making decisions about how to provide midwifery services. However, midwife job satisfaction status in Iran is at an average level, and the officials of the Iranian MOHME can improve this and improve the quality of services provided by this department. Healthcare organizations in other countries can also use multi-dimensional performance assessment systems (e.g. the balanced scorecard) and improve the status of information within the organization through the management accounting unit, and thus improve the quality of midwifery services.

## Footnotes

1. According to unofficial statistics, >50 000 midwives in Iran are working in the private and public sectors.

2. It should be noted that the questionnaire on the quality of midwife services from the perspective of pregnant mothers is collected in the hospitals where the first part of the questionnaire is distributed and collected.

## Reflexivity statement

The authors include two females and two males and span multiple levels of seniority. While three of the authors specialize in performance assessment of healthcare organizations the fourth is an expert in midwifery in Iran. All four authors have extensive experience of conducting qualitative fieldwork in Iran, especially in healthcare organizations.

## Appendix 1. Research questionnaires

### Part 1: cover letter sent to respondents (the first part of statistical population)

Dear respondent

The attached questionnaire has been prepared with the aim of collecting the required information for research entitled ‘Assessing midwifery services in Iran via the balanced scorecard framework’. Since the statistical population consists of all midwives, specialist doctors, nurses and managers who may play a role in the birth process of babies (consultation stages during pregnancy, childbirth, and postnatal counselling and measures) in any manner in the governmental hospitals in Iran, your information is very important to validate the research findings.

This questionnaire is purely scientific and academic and the information contained in it is considered completely confidential and its results will be reported only in the statistical formats of the research. For this reason, mentioning your name and other personal details has not been requested. However, if you wish to receive a summary of the research results by post or email, please complete the information in the section below.

First and last name: ………………………… email: …………………………

Address: ………………………………………………………………………..

The survey will take approximately 20 minutes to complete. Please take your time and answer the questions honestly. We appreciate your time in completing this survey. Should you have any further questions or comments, please feel free to contact me at [email address] or [phone number].

Many thanks

### Survey questions

***General questions***

1. Gender: male □ female □
2. Age (years): <30 □ 30–40 □ 41–50 □ >50 □
3. Educational degree: associate degree □ Bachelor’s □ Master’s □ PhD □
4. Work experience (years): <5 □ 5–10 □ 11–20 □ >20 □

### Specialized questions

*A) The status of intra-organizational information in midwifery services*

1. Management accounting system information is provided in a timely manner to manage midwifery services.
2. The amount of management accounting information in hospital is sufficient to manage midwifery services.
3. Management accounting information is relevant for decisions related to midwifery services.
4. In my organizational unit, there is detailed information about the cost of midwifery-related services.
5. In my organizational unit, complete information for the management of midwifery services is provided to the senior management.
6. In my organizational unit, sufficient information is available to middle and operational management to manage midwifery services.
7. Management accounting information plays an important role in participation of senior managers in planning midwifery services.
8. The operation of the management accounting information system of my organization improves the participation of middle and operational managers in developing the organization’s strategic plan for midwifery services.

*B) The role of the management accounting system in evaluating the performance of midwifery services*

### B-1) Financial aspect

1. In my organization, the strategic management accounting information system provides the necessary information to evaluate the financial performance of the midwifery department.
2. In my organization, the strategic management accounting information system provides the necessary information to estimate all types of medical expenses of the midwifery department.
3. In my organization, the strategic management accounting information system provides the necessary information to evaluate the performance of the midwifery quasi-commercial sectors.
4. In my organization, the strategic management accounting information system provides the necessary information to optimize the best treatment methods (considering cost and effectiveness).

### B-2) Customers aspect (patients)

1. In my organization, the strategic management accounting information system provides the necessary information to determine the level of satisfaction of pregnant mothers.
2. In my organization, the strategic management accounting information system provides the necessary information to identify the needs of pregnant mothers and new-borns.
3. In my organization, the strategic management accounting information system provides the necessary information to make decisions about the discharge schedule of pregnant mothers who have given birth.
4. In my organization, the strategic management accounting information system provides the necessary information for the necessary follow-up of pregnant mothers in the postpartum period.

### B-3) Learning and growth aspect (employee)

1. In my organization, the strategic management accounting information system provides the necessary information to determine the level of satisfaction of midwives and other people working in the midwifery department.
2. In my organization, the strategic management accounting information system provides the necessary information to make decisions about the learning and growth of midwives and other people working in the midwifery department.
3. In my organization, the strategic management accounting information system provides the necessary information for the training programmes and implemented courses and required courses regarding midwives and other people working in the midwifery department.
4. In my organization, the strategic management accounting information system provides the necessary information to apply the incentive and punishment system for midwives and other people working in the midwifery department.

### B-4) Intra-organizational processes aspect

1. In my organization, the strategic management accounting information system provides the necessary information to improve the existing processes in the midwifery department.
2. In my organization, the strategic management accounting information system provides the necessary information to expand the necessary innovations in the midwifery sector.
3. In my organization, necessary software changes adapt to prepare and present strategic management accounting information regarding performance evaluation of midwifery services.
4. In my organization, necessary computer programs to prepare and provide strategic management accounting information regarding performance evaluation of midwifery service exist.

### C) Midwife job satisfaction

*C-1: Job nature*

1. My Job is satisfactory.
2. My job has variety.
3. My job is respected.
4. My job is done to the best of my ability.
5. My job’s nature needs hard working.
6. My job gives me a sense of competence and worth.

### C-2: Salary

1. My salary covers my living expenses.
2. My salary is as much as I deserve.
3. My salary is quite favourable.
4. My salary is higher than my colleagues in similar position.
5. The payment of special benefits is fair.
6. The amount of my benefits is in line with my life needs.

### C-3: Supervisor

1. My supervisor is knowledgeable and expert in his work.
2. My supervisor is honest and upright.
3. My supervisor is an influential person.
4. My supervisor praises good work.
5. My supervisor informs me about the result of the work.
6. My supervisor gives the necessary authority to the employees to perform their duties.

### C-4: Promotions

1. In my job there are good opportunities for promotion.
2. In my job promotions happen regularly.
3. In my job promotions are fair.
4. In my job promotion is done according to the merit of the person.
5. In my job promotion is a good opportunity for advancement.
6. In my job promotions are made with the aim of improving quality.

### C-5: Colleagues

1. My colleagues are hardworking and diligent.
2. My colleagues are with Frost.
3. My colleagues feel responsible.
4. My colleagues keep each other’s secrets.
5. My colleagues respect each other’s privacy.
6. My colleagues motivate me.

### Part 2: cover letter sent to respondents (the second part of statistical population)

Dear respondent

The attached questionnaire has been prepared with the aim of collecting the required information for research entitled ‘Quality of services, intra-organizational information, job satisfaction and performance assessment of midwifery services in Iran via the balanced scorecard framework’. Since the statistical population includes mothers who recently gave birth in Iran’s public hospitals, your information is very important to validate the research findings.

This questionnaire is purely scientific and academic and the information contained in it is considered completely confidential and its results will be reported only in the statistical formats of the research. For this reason, mentioning your name and personal details has not been requested. However, if you wish to receive a summary of the research results by post or email, please complete the information in the section below.

First and last name: ………………………… email: …………………………

Address: ………………………………………………………………………..

The survey will take approximately 10 minutes to complete. Please take your time and answer the questions honestly. We appreciate your time in completing this survey. Should you have any further questions or comments, please feel free to contact me at [email address] or [phone number].

Many thanks

### Survey questions

***General questions***

1. Age (years): less than 25 □ 25-35 □ 36-45 □ over 45 □
2. Educational degree: associate degree and others □ Bachelor’s □ Master’s □ PhD □

### Specialized questions

*Quality of midwifery services*

1. Hospital officials pay attention to the needs of pregnant mothers before giving birth.
2. I am satisfied with respect to personal and collective hygiene in the midwifery department.
3. Obstetrics specialist doctors pay good attention to the health of mothers and babies.
4. Midwives and nurses in the midwifery department pay good attention to the health of mothers and babies.
5. The equipment of the midwifery department is in good condition.
6. The provided services in the midwifery department are up-to-date.
7. In terms of service delivery time, the midwifery department is in a good situation.
8. In general, I am satisfied with the services provided in the midwifery department of the hospital (before, during and after delivery).
